# The Global Burden of Occupational Disease

**DOI:** 10.1007/s40572-017-0151-2

**Published:** 2017-07-21

**Authors:** Lesley Rushton

**Affiliations:** Department of Epidemiology and Biostatistics, Imperial College London, MRC-HPA Centre for Environment and Health, Norfolk Place, London, W2 1PG UK

**Keywords:** Occupation, Cancer, Burden estimation, Respiratory disease, Exposure assessment, Impact

## Abstract

**Purpose of Review:**

Burden of occupational disease estimation contributes to understanding of both magnitude and relative importance of different occupational hazards and provides essential information for targeting risk reduction. This review summarises recent key findings and discusses their impact on occupational regulation and practice.

**Recent Findings:**

New methods have been developed to estimate burden of occupational disease that take account of the latency of many chronic diseases and allow for exposure trends and workforce turnover. Results from these studies have shown in several countries and globally that, in spite of improvements in workplace technology, practices and exposures over the last decades, occupational hazards remain an important cause of ill health and mortality worldwide.

**Summary:**

Major data gaps have been identified particularly regarding exposure information. Reliable data on employment and disease are also lacking especially in developing countries. Burden of occupational disease estimates form an important part of decision-making processes.

## Introduction

Estimation of the burden of disease from different risk factors is a useful public health tool in assessing premature deaths and illness. A range of burden measures, such as disease proportions, numbers of deaths, incidence or prevalence of diseases and quality of life measures, provide decision makers with data to facilitate prioritisation of risk reduction strategies. Many studies to date have focused on lifestyle causes of illness but various environmental and occupational risks are now increasingly being included.

It is generally accepted that the working environment should not present a risk of injury or disease but many thousands of workers worldwide remain exposed to hazardous substances both in the developed world and particularly in countries that are rapidly industrialising. Diseases and their risk factors that rarely if ever occur in non-occupational settings such as silicosis and asbestosis continue to be a problem in these countries.

Is occupation important in the global picture of the burden of disease and is estimating the burden of occupational disease achievable and worthwhile? This review summarises briefly the methods commonly used for estimating burden of occupational disease and gives an overview of some of the more recent developments. Results from some key studies published in recent years are reviewed. The impact of some of these studies in raising awareness of occupationally related disease, on regulation and on changing practice in the workplace is highlighted. The future, or not, of burden estimation is also discussed.

## Methods of Estimating Occupational Burden of Disease

The measure most often used in burden studies is the population attributable fraction (PAF) i.e. the proportion of a disease that would be avoided in the absence of the risk factor under consideration. This measure then allows derivation of others such as numbers of cases, deaths etc. There are a number of methods that have been used for estimating the PAF. These include approaches such as:

- The use of the Delphic principle [1] which uses panels of experts to estimate attributable fraction [2]
- Linkage of national databases such as census and cancer registry data [3]
- Descriptive studies of incidence or deaths [4]
- Use of absolute risk when the occupational exposure is considered the only or major cause e.g. asbestos and mesothelioma[5]

The most common method, however, is to estimate the PAF by combining a risk estimate for the disease associated with the agent of concern with the proportion exposed to the agent in the population of interest using an appropriate equation [6]. Some form of Levin’s equation [7] is generally used if the risk estimate is from published studies and the population exposed is from independent national data; Miettinen’s equation is appropriate [8] if both data components are from the same study e.g. a population-based case-control study. Several studies in different countries have estimated PAFs for occupationally related cancer using a variety of methods. Burden estimates range between 3 and 10% partly due to differences in the numbers of cancers and carcinogens considered [9–16].

## The British Study

### Current Burden Estimation

The British occupational burden of cancer study developed a structured approach to evaluating the burden of occupationally related cancer in Britain. The study considered all carcinogens and cancer sites classified by the International Agency for Research on Cancer (IARC) as definite (group 1) or probably (group 2A) occupational human carcinogens (over 40 carcinogens and 20 cancer sites were included). Cancer latency was taken into account by defining a risk exposure period (REP) as the exposure period relevant to a cancer appearing in the year of burden estimation; 10–50 years was used for solid tumours and 0–20 years for haematopoietic cancers. The proportion ever exposed at work over the REP was then estimated using national data sources, accounting for employment turnover and life expectancy, and adjusted for changes in employment patterns [17]. The risk estimates were obtained from published literature using expert judgement to select studies where the patterns of exposure and potential confounders such as smoking paralleled those of Britain. Disability-adjusted life-years (DALY), the sum of years of life lost (YLL) (estimated from cancer deaths and life expectancy) and years lived with disability (YLD) were also estimated.

**Fig. 1:**
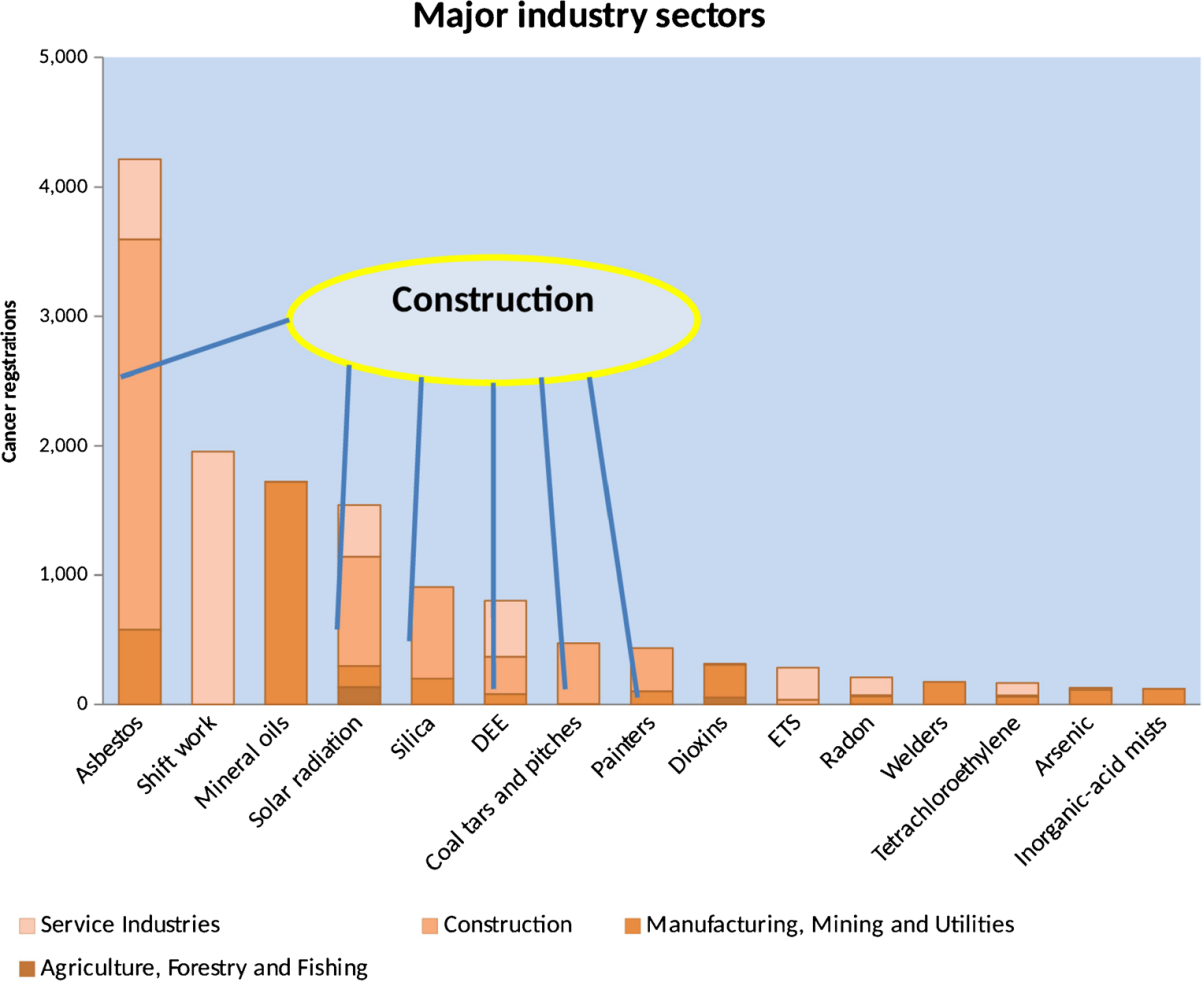
Numbers of occupationally related cancer registrations (2011) by carcinogen and major industry sector

Overall, the PAF was about 5% (higher in men, 8% than women, 2.3%) giving over 8000 attributable cancer deaths (2005) and approximately 13,500 newly occurring cancers (cancer registrations) [18]. Figure 1 illustrates the cancer incidence figures for the top 15 carcinogens and the distribution among the 4 broad occupational groups. The industry of most concern is construction with 56% of the total attributable cancers in men occurring in this industry. For women, 54% of the total attributable cancers were attributable to shift work (breast cancer). Unlike the majority of carcinogens associated with cancers in men which are classified IARC group 1 definite human carcinogens, shift/night work is currently an IARC group 2A carcinogen, a probable human carcinogen.

The British team have recently extended their methods to estimate PAF by age group for the example of melanoma associated with occupational exposure to solar radiation [19]. This takes account of variation in cancer rates by age; the exposed population and national working population have been assumed to have the same age structure and workers were assumed to enter the workforce between ages 15–45 and retire at 65. Melanoma was not estimated in the earlier project because of uncertainty in the relative contributions of leisure and occupational sun exposure to melanoma risk. The recent literature is still somewhat equivocal. However, it seems plausible that work exposure should contribute to the overall risk. Assuming causality therefore, the estimated PAF for melanoma associated with sun exposure at work was 2.0% giving 48 deaths and 241 cancer registrations (newly diagnosed melanomas) and an average years of life lost (YLL) through early death of about 17 years. Once again construction was the main industry of concern with nearly half of the deaths and newly diagnosed melanomas followed by agriculture, public administration and defence and land transport. Over half of the melanomas occurred after retirement age (65+), highlighting the fact that the long latency of occupational cancers may lead to the cancer occurring many years after leaving work.

### Extension to Economic Costs

These burden estimates and in particular the quality of life results have been used by economists from the Health and Safety Executive (HSE) to quantify the economic cost of exposures to workplace carcinogens in Britain [20•]. Total economic costs to society of new cases of work-related cancer in Britain in 2010, arising from past working conditions, are estimated as approximately £12.3 billion with the three major cancers being lung (£6.8 billion), mesothelioma (£3.0 billion) and breast (£1.1 billion). Individuals bear almost all (£12.0 billion, 98%) of the costs of work-related cancer due largely to ‘human’ costs—a monetary value on the effects of cancer on quality of life or loss of life for fatal cancers (£11.4 billion). By comparison, only £461 million is borne by employers. The authors point out that the work-related cancers often occur after retirement due to the long latency between occupational exposure to carcinogens and development of many cancers. Thus, employers do not incur costs such as disruption from sickness absence and paying sick pay.

### Predicting Future Burden

Estimation of the current burden for different occupational carcinogens provides valuable data for decision makers. However, prediction of the potential effectiveness of reduction programmes is also desirable. To this end, the British team extended their methodology to forecast the burden associated with the carcinogens shown in Fig. 1 at several time points in the future for a range of scenarios, such as the introduction of new occupational exposure limits, increased levels of compliance with these limits and other reductions in worker exposure. Risk exposure periods were projected forward in time with the contribution of past exposure to future cancer risk decreasing as time points increased. Adjustments were made for predicted employment turnover and life expectancy, and adjusted for changes in employment patterns as in the current burden methods [21]. Without intervention, occupational attributable cancers were forecast to remain at over 10,000 annually by 2060 [22]. Effective interventions were shown to be reduction of workplace exposure limits and in particular improving compliance with these limits.

## Global Burden of Occupational Disease

Although estimation of occupationally related mortality and morbidity in single countries may be achievable, attempts to do this globally have faced enormous challenges of data availability, quality and collation. Not least is the issue of exactly what constitutes an occupational risk and which of these and their associated diseases is it feasible to include. Estimates of the global burden of occupational disease thus vary although a general conclusion is that the problem is a major one and probably most estimates are under-estimates.

The International Labour Organisation and the World Health Organisation have both been key players in the effort to enumerate this issue; both organisations update their estimates at regular intervals. They arrive at similar estimates of 5–7% of global fatalities attributable to work-related illnesses and occupational injuries [23, 24]. Takala et al. 2012 provide an overview of data on employment and occupational mortality and morbidity, publically available literature and reports on occupational burden of disease [25]. They estimate that globally there are 2.3 million occupationally related deaths each year attributable to work with the majority, 2.0 million, being due to occupational diseases. Overall, cancer forms the largest component (32%) followed by work-related circulatory diseases (23%), communicable diseases (17%) and occupational accidents (18%) with the latter two being far more prevalent in developing and rapidly industrialising countries. For cancer, this translates to 660,000 deaths with asbestos being the exposure contributing the largest proportion [26]. Another International Labour Organisation publication gives information on non-fatal occupational accidents and fatal occupational cancers. Over 313 million non-fatal occupational accidents (with at least 4 days absence) in 2010 are estimated, and over 666,000 fatal occupationally related cancers [27].

**Fig. 2:**
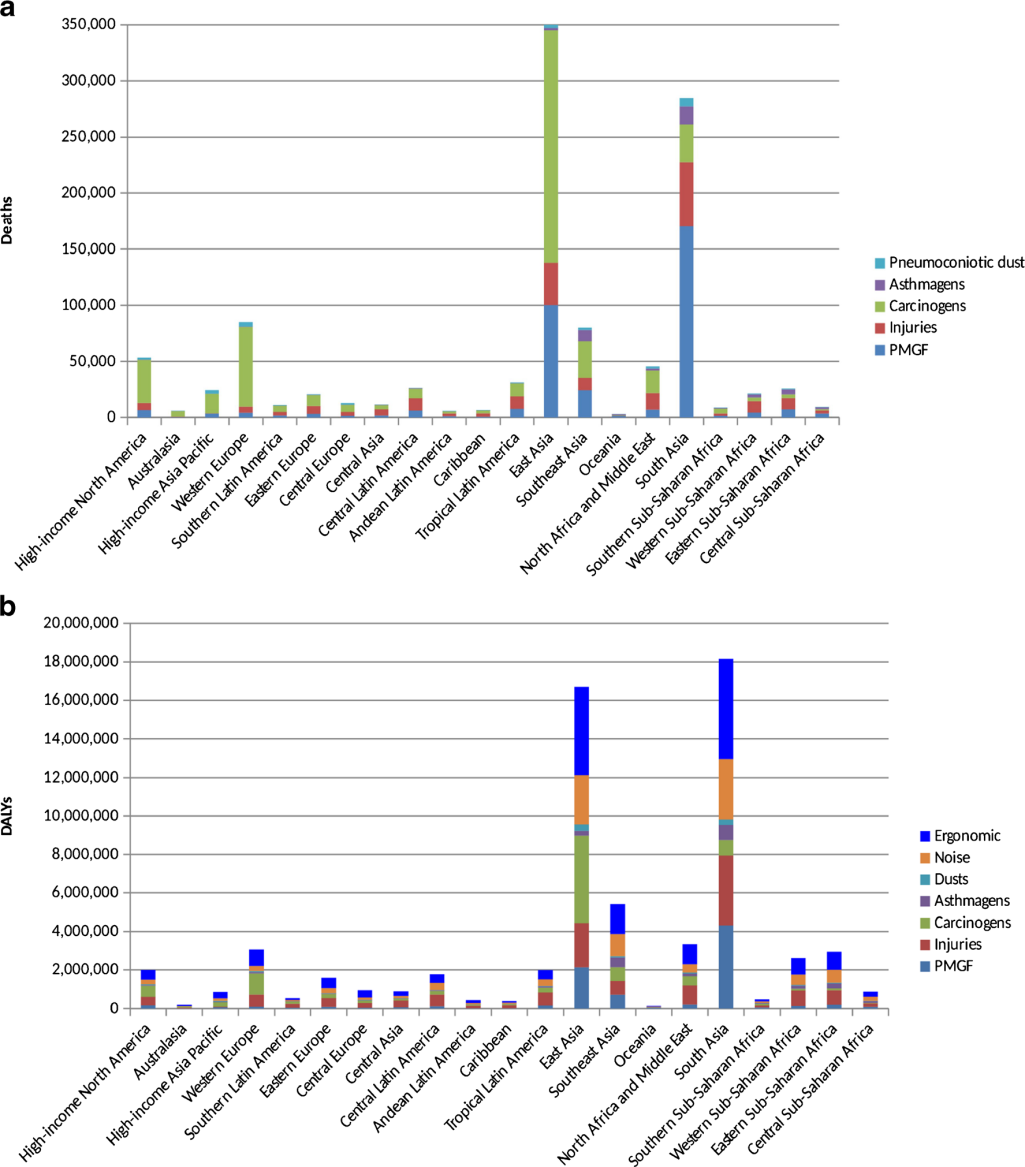
**a** Numbers of global occupationally related deaths in 2015 by WHO region and major disease group (This figure was derived from data available on the following site: GBD Compare. IHME, University of Washington; 2016. https://vizhub.healthdata.org/gbd-compare/. Accessed January 2017.). **b** Numbers of global occupationally related DALYS in 2015 by WHO region and major disease group. (This figure was derived from data available on the following site: GBD Compare. IHME, University of Washington; 2016. https://vizhub.healthdata.org/gbd-compare/. Accessed January 2017)

The WHO has conducted Comparative Risk Assessments (CRA) to estimate burden of disease as part of the Global Burden of Disease project; this provides comprehensive descriptive data, including trends, in mortality and morbidity from major diseases, injuries and risk factors. Several estimates have been published since 1990 [28] and 2010 [29], with the 2010 GBD study updated in 2013 [30] and 2015 [31]. To attempt to ensure consistency worldwide, the GBD project has stringent data requirements and this has thus limited the risk factors evaluated and in particular for occupation which has been included since 2000. The first occupational estimates included only lung cancer (8 lung carcinogens), mesothelioma and leukaemia (3 leukemogens) and estimated globally that there were 152,000 deaths annually from occupational cancers (lung cancer 102,000, leukaemia 7000, mesothelioma 43,000) and nearly 1.6 million DALYs mainly originating from lung cancer and mesothelioma [32]. The most recent update (2015) incorporated and extended the methods used by the British occupational cancer burden study and included 14 carcinogens (IARC group 1) (compared with 30 group 1 carcinogens and 10 group 2A carcinogens in the British study) related to seven cancer types—kidney (trichloroethylene), lung (arsenic, asbestos, beryllium, cadmium, chromium VI, diesel engine exhaust, second-hand smoke (from tobacco smoking) (SHS), nickel, polycyclic aromatic hydrocarbons (PAHs), silica), larynx (asbestos, strong inorganic-acid mists), leukaemia (benzene, formaldehyde), mesothelioma (asbestos), nasopharynx (formaldehyde) and ovary (asbestos). In addition, occupational burden was estimated for asthmagens, particulate matter, gases and fumes (PMGF), noise, ergonomic risk factors for low back pain, and risk factors for occupational injury [33]. 1,086,000 deaths were estimated to occur globally due to occupational risks. These include 489,000 occupationally related cancer deaths with important causes being asbestos (180,000), diesel engine exhaust (120,000), silica (86,000) and SHS at work (96,000); SHS has largely been banned in the workplace in many developed countries but still remains a major issue in developing and rapidly industrialising countries. Occupational exposure to asthmagens is estimated to cause 42,000 deaths, with PMGF causing 357,000 (mainly COPD) and workplace injuries causing 204,000 deaths. Figure 2a shows the distribution of deaths by cause across the 21 WHO regions [34•].

Occupational risks are ranked eleventh in terms of total DALYs for men and thirteenth for women, with dietary risk ranked top for both, followed by smoking for men and child and maternal malnutrition for women and high systolic blood pressure for both. Total occupational DALYs are 63,600 approximately with low back pain contributing 18,400 followed by occupational injuries 13,500, noise 10,900 and occupational carcinogens 9800. These DALY results highlight the huge burden worldwide caused by occupationally-related morbidity. In many countries the leading cause of sickness absence is musculoskeletal disorders. Figure 2b shows the DALYs by WHO region.

## Ongoing Work Around the World

### Canada

The Canadian study ‘the human and economic burden of cancer in Canada’ is a collaborative study of Canadian researchers. The study has extended and expanded the methods developed by the British team to use much more detailed exposure data about Canadian workplace exposure to carcinogens including number exposed and also exposure measurements. A major component is the use of the burden of occupational cancer estimates in combination with information on costs of health care and administration, informal caregiving, output and productivity losses and health-related quality life losses to estimate the economic burden of newly diagnosed occupational cancers. This includes all current and future costs incurred by afflicted workers, their families, communities, employers and society at large. Throughout the study period, the research team has actively engaged with funders and stakeholders [35]. Preliminary results have been discussed and final results are expected in the near future.

### USA

The USA is included as one of the WHO regions analysed in the GBD study. However, there is no comprehensive study of the current occupational disease burden due to past exposures for the USA. Purdue et al. [36] reviewed published estimates of occupational cancer burden but identified only 2 for the USA that estimated current burden, the Doll and Peto study of 1981 (4%) [9] and a later paper by Steenland et al. [11] (2.4–4.8%) [11]. For the examples of bladder and lung cancers due to work as a painter and shift (night) work associated with female breast cancer, Purdue et al. used the methods from the British study to demonstrate the potential usefulness of burden estimation for informing prevention. Work as a painter accounted for a very small proportion of cancers of the bladder and lung in 2010, with PAFs of 0.5% for each site. The results for shift work contrasted with this giving a PAF of 5.7%, translating to 11,777 attributable breast cancers.

### Australia

An estimate of the burden of occupational cancer in Australia by Fritschi and Driscoll [37] applied Finnish estimates of the proportion of cancers caused by occupation to numbers of cancers in Australia and applied European Union estimates of the proportion of workers exposed to carcinogens to Australian industrial data [37]. They estimated that about 5000 incident cancers each year were due to occupation (11% males, 2% females) with about 34,000 occupationally related non-melanoma skin cancers. In addition, they estimated that there were about 1.7 million workers in Australia occupationally exposed to carcinogens.

To provide more objective figures on the numbers of Australians exposed to workplace carcinogens, the Australian Work Exposures Study (AWES) surveyed a random sample of just over 5000 currently employed men and women and interviewed them by telephone about their current job [38]. A web-based application (OccIDEAS) [39] was utilised in which participants were asked about their job tasks and predefined algorithms were then used to automatically assign exposures. 37.6% were assessed as being exposed to at least one occupational carcinogen in their current job suggesting that 3.6 million (40.3%) current Australian workers could be exposed to carcinogens in their workplace. Exposure prevalence was highest among farmers, drivers, miners and transport workers.

The Australian team then developed a lifetime risk approach to predict how many workers exposed now would develop a future cancer. This addresses burden from a different perspective than the more commonly used PAF approach which estimates the burden now from exposure in the past. The lifetime risk approach is useful if information on past exposure is scarce; however, it requires prediction of future general population disease risk which is a potential source of inaccuracy. The ‘future excess model’ developed by Fritschi et al. [39] estimates the lifetime excess fraction due to a workplace carcinogen exposure and is based on disease-free population’s person-years rather than the total population, takes account of age-specific survival and uses a risk estimate obtained from the literature for each carcinogen and its associated cancer site. It should be noted that the future excess fraction is not directly comparable with the PAF [40].

It was estimated that out of the Australian working population who were exposed to occupational carcinogens in 2012, the number of occupationally related cancers that would develop over the period to 2094 would be 68,500 (1.4%), with the majority being lung cancers (26,000), leukaemias (8000) and malignant mesotheliomas (7500) [41].

## Impact of the Occupational Burden Studies

Burden estimation studies have the potential to influence policies that effect improvement in health of workers. The HSE have used the information from the British cancer burden study to inform the development of their health strategies, in their guidance documents and to identify practical interventions together with stakeholders. The results have been similarly used to make workers aware of the issue of occupational disease and cancer by worker organisations such as the Trade Union Council and have underpinned a major campaign, the ‘No time to lose’ launched by the Institution for Occupational Safety and Health (IOSH) [42]. The campaign provides free practical, original materials to businesses to help them deliver effective prevention programmes and encourages them to sign up to committing to reduce exposure to carcinogens.

There is increasing interest in evaluating the financial impact of work-related disease and cancer in particular on both workers and industry and incorporating this into decision-making. The European Union (EU) have used results from a socio-economic health and environmental impact assessment of introducing binding occupational exposure limits (OEL) for 25 work place carcinogens to inform, together with other data, changes to EU legislation. Methods from the British cancer burden study were extended to estimate health costs and benefits of introduction of up to three different OELs compared to no intervention [43]. Compliance costs were separately estimated and a cost-benefit ratio was calculated. The cost-benefit results have made a strong impact on the decision-making process although for a few carcinogens the proposed OEL is greater than that based on a health-based risk assessment arrived at by one of the EU Scientific Committees [44]. Greater health benefits gained from lower values can be outweighed in the decision-making process if there is thought to be a ‘disproportionate’ cost to certain sectors of industry, for example, small and medium-sized enterprises, even if these are the very ones where higher exposures are expected to be found.

## Discussion and Conclusions

This paper has provided an overview of the findings from some of the projects estimating the burden of disease from exposure to workplace hazards. There is a burgeoning research industry in this area and several ongoing studies will be reporting their results in the next few years. These include the Canadian study described earlier, more detailed results on occupation from the Global Burden of Disease 2015, a study in the Singapore construction industry, a large project in France estimating the burden of disease from many risk factors including occupation led by IARC and an EU OSHA study to evaluate the economic burden of exposure to occupational carcinogens in the EU. Many of them have extended and improved existing methodology to address data inadequacies and assumptions and also important country-specific circumstances, for example if a large proportion of the workforce is non-resident and/or transient.

Data limitations and assumptions made lead to uncertainties about the ‘true’ magnitude of the results. Random error confidence intervals go some way to expressing the uncertainty but do not address potential uncertainty sources such as: assumptions about cancer latency and thus the length of the relevant exposure window before cancer development; lack of data on the proportions exposed at different exposure levels within industry sectors or jobs; choice of the risk estimates and whether the studies from which these are chosen are compatible with the population of concern regarding exposures, confounders etc.; use of risk estimates from studies of occupational groups affected by the healthy worker effect i.e. a reduced overall risk estimate compared to the general population leading to an underestimation of the true effect and thus an underestimation of the burden; methodological issues such as the use of Levin’s equation with adjusted risk estimates and employment turnover methodology. Credibility intervals exploring the relative contributions of important sources of uncertainty have shown that the choice of relative risk and the employment turnover estimates contribute most to overall estimate uncertainty with bias from using an incorrect estimator making a much lower contribution [45].

The magnitude of the overall burden depends on which diseases and risk factors and how many are included. An update of an overall cancer burden estimate to include additional carcinogens, for example as a result of further agents being classified as definite human carcinogens by IARC, will generally give a higher overall AF and the relative importance of each agent included previously will potentially change. Changes in the relative importance may also occur when an intervention is introduced and an individual risk has been reduced. In a non-occupational setting, the incidence of sudden infant death syndrome (SIDS) greatly reduced after a campaign to encourage parents to lay their babies on their sides or back. However, the relative importance of smoking and bottle feeding then became more apparent [46]. Thus, caution is required when interpreting the absolute numbers from burden of disease studies although these can serve to emphasise the importance of a particular risk factor or effect on a certain disease group. Of particular use, however, is the use of ranked relative measures by disease, exposure, industry etc.

How much more burden estimation should we do and how often should we repeat the exercise? Compared with occupational cancer burden studies, there are far fewer focusing on cardiovascular disease (CVD). Estimates from 6 previous studies range from 1 to 23% but cover different outcomes e.g. CVD overall, ischaemic heart disease, acute myocardial infarction, hypertension etc. and different combinations of potential exposures e.g. noise, low job control, shiftwork, SHS, psychosocial stress etc. [11, 12, 47–50]. There is, however, a substantial literature on work-related factors that might increase risk of various CVD outcomes. In addition to the above, exposures investigated include sedentary work, noise, heat, irradiation and a number of chemicals including lead, cobalt, arsenic, carbon monoxide, solvents etc. Like many cancers, there are also well-established personal characteristics and lifestyles that contribute to increased CVD risk which need taking into account. However, unlike cancer, there is no well-respected and established system of classification of occupational hazards for CVD. Estimation of occupationally related CVD thus presents methodological and data challenges which would benefit from future research.

The GBD project has a programme of regularly updating and revising their estimates, sometimes every 1 or 2 years. This may cause confusion among potential users especially if numbers are very different without obvious reasons e.g. if methods have been revised. If the status quo remains the same then perhaps resources might be better used in filling some of the gaps identified in these studies. Individual countries wishing to carry out their own burden estimation may also find that they can fairly easily adapt either existing methods and/or existing data such as risk estimates or even PAFs.

Major data gaps exist particularly regarding lack of exposure measurement data and inadequate national information on employment, numbers of exposed individuals and the levels to which they are exposed. In addition, in many countries, occupation is not a mandatory item in routinely collected health data such as family doctor and hospital records and cancer registrations. A global effort to fill this gap would facilitate evaluation of occupational risks and allow use of techniques such as application of job exposure matrices.

The use of burden of occupational disease estimates by the EU to set occupational exposure limits has highlighted the fact that decision makers will use these figures together with other major information on costs, benefits, numbers exposed, countries and size of industries involved, and toxicological risk assessments. The opinion of members of one or more expert committees will also influence final decisions. Further development of methods to predict how future burden can be impacted by different interventions and validation of these with data for example from exposure monitoring would be valuable.

In conclusion, burden estimation is an important public health tool to inform risk reduction strategies and the prevention of disease caused by workplace exposures.
